# Soil pH Determining the Assembly Processes of Abundant and Rare Bacterial Communities in Response to Cultivation Modes in Lemon Farmlands

**DOI:** 10.3390/plants14121852

**Published:** 2025-06-16

**Authors:** Hao-Qiang Liu, Si-Chen Li, Hong-Jun Li, Zhu-Chun Peng

**Affiliations:** 1 Citrus Research Institute, Southwest University, Beipei District, Chongqing 400715, China; lisichencitrus@126.com (S.-C.L.); lihongjun@cric.cn (H.-J.L.); pengzhuchun@cric.cn (Z.-C.P.); 2 National Engineering Research Center for Citrus, Chinese Academy of Agricultural Sciences, Beipei District, Chongqing 400712, China

**Keywords:** environmental adaptation, distance–decay of similarity, greenhouse, phylogenetic signal, stochastic assembly

## Abstract

Here, the biogeographic patterns of abundant and rare bacterial taxa in lemon farmlands with different cultivation modes were examined using the dataset obtained from high-throughput sequencing. The abundant sub-communities exhibited a lower richness, a similar abundance proportion, and lower compositional variations than rare taxa. With regard to different cultivation modes, a lower richness but higher beta-diversity distance was observed in abundant bacterial taxa from greenhouse soils compared to other open field farmlands. In addition, some potential indicators, including Proteobacteria, Chloroflexi, and Bacteroidota, were found to be enriched in the abundant sub-communities in greenhouse soils. Moreover, a stronger environmental-related distance–decay of similarity was observed in abundant taxa from greenhouse soils, but in hilly-converted farmlands for rare taxa. The abundant sub-communities were more sensitive to environmental changes and more tightly phylogenetically clustered. In contrast, homogeneous selection dominated the assembly of rare taxa, which was insensitive to dispersal limitations. Soil pH was identified as the key factor to driving the assembly of soil bacterial communities, with a more deterministic and stochastic assembly for abundant and rare taxa, respectively, at the neutral environments.

## 1. Introduction

The microbial community is typically composed of a small number of highly abundant taxa and a large number of rare taxa with extremely high diversity [1]. Differentiating between abundant and rare sub-communities is crucial for understanding the structure and function of microbial communities [2]. Abundant and rare microbial taxa exhibit distinct patterns in community diversity and taxonomic composition [3]. Abundant taxa account for a small proportion of the total number of taxa, but represent a large proportion of the overall community abundance [4]. In contrast, rare microbial taxa contribute significantly to the biodiversity of ecosystems with a low abundance [5]. Abundant and rare taxa also differ markedly in their ecological roles in the relationships between biodiversity and ecosystem functions [6]. Abundant taxa are often considered the most important for core ecosystem functions due to their high abundances [7], and changes in the abundance of these taxa can drive variations in these broad functional measures [8]. For rare taxa, studies have shown that these taxa, despite their low abundance, can have disproportionately large effects on ecosystem processes and stability [9]. In addition, rare taxa generally provide functional redundancy, which is crucial for maintaining ecosystem processes under changing environmental conditions, as rare taxa can potentially replace functions lost if dominant taxa are diminished due to environmental stresses [10]. By comparing them with abundant taxa, researchers can better understand the overall biodiversity and how these different components contribute to ecosystem resilience and function [11].

In addition, the dynamics between abundant and rare taxa can determine the resilience of ecosystems to environmental changes [12]. Understanding how these groups respond differently to environmental stresses can inform the conservation strategies and management practices of ecosystems [13]. Abundant taxa tend to have wider niche breadths and can better adapt to environmental changes compared to rare taxa, allowing them to thrive under changing conditions [14]. Understanding how abundant taxa respond and adapt to environmental changes is crucial for predicting ecosystem-level responses [15]. On the other hand, rare taxa often show different responses to environmental changes compared to abundant taxa [16]. Rare taxa might serve as a reservoir of functional capabilities that become crucial under specific environmental conditions [17]. For instance, in response to environmental stressors or changes, these rare taxa can increase in abundance and take over functions necessary for ecosystem recovery and maintenance [18]. Moreover, rare microbial taxa can act as indicators of ecosystem health and shifts in environmental conditions [19]. Their presence or absence can provide early warning signs of ecological changes that might not be detectable by examining only the abundant taxa [20]. By understanding these contrasting responses, researchers can better predict how microbial communities as a whole will respond to environmental perturbations, and how the balance between abundant and rare taxa may shift [21]. This knowledge is crucial for managing and conserving ecosystem functions in the face of global change.

Moreover, comparing rare and abundant taxa helps refine ecological theories and models about community assembly within ecosystems [22]. The assembly of microbial communities is influenced by various processes such as diversification, dispersal, selection, and drift [23]. These processes could be divided into deterministic and stochastic processes, and their balance drive the biogeography of microorganisms [24]. Environmental filtering, which structures communities based on abiotic factors, and trait clustering due to competitive ability differences or ecological interactions, are key mechanisms of deterministic processes influencing community assembly [25]. These processes are positively related to community alpha diversity, microbial interaction degree, and bacterial predatory-specific gene abundance [26]. On the other hand, stochastic processes are governed by neutral theory and involve random assembly through birth–death, drift, and speciation [27]. Among them, drift is most important when selection is weak, alpha diversity is low, and the total number of community members is small [28]. Accordingly, previous studies indicated that abundant taxa were dominantly shaped by deterministic processes, such as environmental filtering, while the assembly of rare taxa was governed by stochastic processes, such as dispersal limitation [29,30,31]. As their relative importance can vary depending on the environment and the specific microbial community [32,33,34], distinguishing the assembly mechanisms of abundant and rare taxa is crucial for accurately interpreting microbial community dynamics, interactions, and functional potential [35]. This knowledge is valuable for understanding microbial community ecology and its applications.

Agricultural ecosystems provided humans with food, forage, bioenergy, and pharmaceuticals, which are essential for human well-being [36]. As one of the most valuable fruits, lemon cultivation has rapidly developed in China, especially for Chongqing and Sichuan Province in southwest area [37]. In the present study, we aimed to (I) evaluate the effects of cultivation modes on the diversity and composition of microbial communities, (II) explore the differences of environmental adaptation, and (III) recognize the major factors influencing the assembly of abundant and rare bacteria in soils of lemon farmlands. These questions were addressed using the high-throughput sequencing datasets of soil bacteria along with 15 environmental factors in greenhouse and open-field lemon farmlands. Given the unique environmental conditions of greenhouses, we hypothesized that the biogeographic patterns and assembly mechanisms of soil bacterial communities could be different in this cultivation mode compared to other open farmlands in Chongqing, China. Moreover, we also hypothesized that the responses of assembly processes governing abundant and rare bacterial sub-communities could be distinct under environmental changes. Insights gained from this study contribute to the development of ecological theories related to microbial succession, community assembly, and the impact of microbial diversity on ecosystem processes.

## 2. Results

### 2.1. General Patterns of Abundant and Rare Taxa

**Figure 1:**
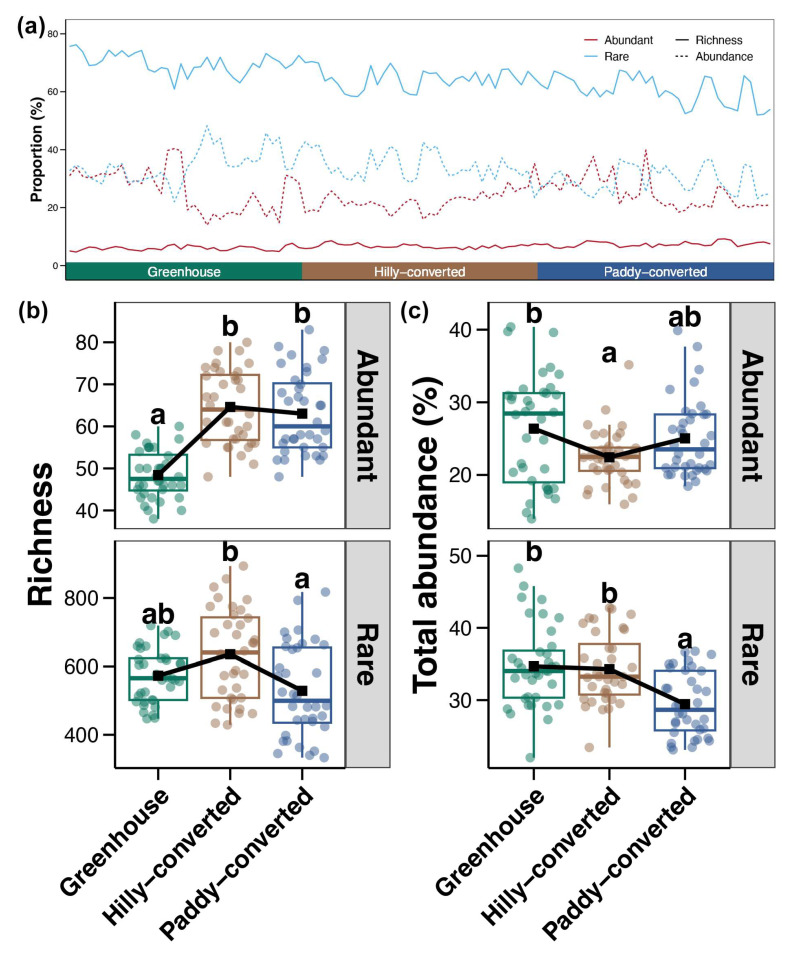
(**a**) The proportions (percentage of whole community) of abundant and rare taxa in richness and abundance, respectively, across studied soil samples. The red and blue lines represent abundant and rare taxa, respectively. The solid and dotted lines represent the proportion based on richness and relative abundance, respectively. (**b**) Differences in the richness of abundant and rare sub-communities among different cultivation modes. (**c**) Variations in the total abundance of abundant and rare taxa among different cultivation modes. Different lowercase letters above each box in the same sub-figure represent significant differences among samples from different cultivation modes (Tukey’s HSD test, *p* < 0.05).

According to the sequencing results, a total of 2,634,985 high-quality reads were obtained from the 108 soil samples, which were clustered into 36,680 ASVs belonging to 48 bacterial phyla and 857 genera. As expected, most of them were identified as rare taxa (34,949, 95.28%) and only 115 ASVs (0.31%) were recognized as abundant taxa. The average richness proportion of abundant and rare taxa in the studied soil samples was 6.67% and 64.91%, respectively, and the average abundance proportion was 24.61% and 32.81%, respectively (Figure 1a). For soils from different cultivation modes, the richness of abundant taxa was significantly higher in soils from hilly- and paddy-converted farmlands compared to those from the greenhouses (Tukey’s HSD test, *p* < 0.05, Figure 1b). The richness of rare taxa was also the highest in hilly-converted samples but significantly decreased in paddy-converted farmlands (Tukey’s HSD test, *p* < 0.05, Figure 1b). The total abundance of abundant taxa in soils was the highest in greenhouse but lowest in hilly-converted farmlands (Figure 1c). In addition, a significantly higher total abundance of rare taxa was found in soils from greenhouse and hilly-converted farmlands than those in paddy-converted farmlands (Tukey’s HSD test, *p* < 0.05, Figure 1c).

### 2.2. Effects of Cultivation Modes on Abundant and Rare Taxa

**Figure 2:**
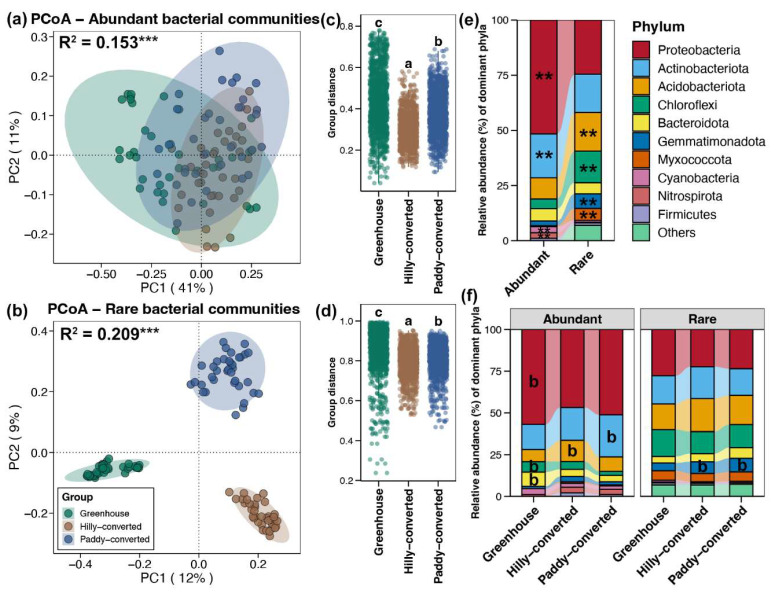
PCoA and adonis test for abundant (**a**) and rare (**b**) sub-communities in soils among different cultivation modes. *** represents the *p*-value of adonis test lower than 0.001. Differences in the Bray–Curtis distance between of abundant (**c**) and rare (**d**) sub-communities, respectively, between different soils from each cultivation mode. Different lowercase letters above each box in the same sub-figure represent significant differences among samples from different cultivation modes (Tukey’s HSD test, *p* < 0.05). (**e**) The average relative abundances of dominant bacterial phyla for abundant and rare sub-communities in all studied soils. ** represents significantly higher relative abundance of bacterial phylum in abundant or rare sub-communities (Wilcox rank-sum test, *p* < 0.05). (**f**) Variations in the relative abundances of dominant bacterial phyla in abundant or rare sub-communities from soils among different cultivation modes. The letter “b” represents significantly higher relative abundance of bacterial phylum in corresponding cultivation mode compared to others (Tukey’s HSD test, *p* < 0.05).

PCoA revealed the differences in the community structure of abundant and rare taxa among different cultivation modes. The distribution of abundant sub-communities among different cultivation modes had a large of overlap (Figure 2a); in contrast, the rare sub-communities were totally separately clustered (Figure 2b). The results of the adonis test also revealed the stronger influences of cultivation modes on rare taxa than that to abundant sub-communities (0.209 vs. 0.153), although both of them were significant (*p* < 0.05, Figure 2a,b). The Bray–Curtis distance of abundant and rare sub-communities between different soils from each cultivation mode were further compared, respectively. Both of them showed the highest intra-variation in greenhouses, followed by paddy-converted farmlands, and the lowest in hilly-converted farmlands (Tukey’s HSD test, *p* < 0.05, Figure 2c,d).

For both abundant and rare taxa, Proteobacteria was the most dominant bacterial phyla, following by Actinobacteriota, Acidobacteriota, and Chloroflexi, but their proportion in abundant or rare taxa were totally distinct (Figure 2e). Abundant sub-communities contained a higher proportion of Proteobacteria, Actinobacteria, Cyanobacteria, and Nitrospirota, while the proportion of Acidobacteriota, Chloroflexi, Gemmatimonadota, and Myxococcota were higher in rare taxa (Wilcox rank-sum test, *p* < 0.05, Figure 2e). In addition, their variations among different cultivation modes were also compared. The results showed higher abundances of Proteobacteira, Chloroflexi, and Bacteroidota in abundant sub-communities from greenhouse soils, with more abundant Acidobacteriota and Actinobacteriota in hilly- and paddy-converted farmlands, respectively (Tukey’s HSD test, *p* < 0.05, Figure 2f). In contrast, only Gemmatimonadota was found to be enriched in the rare sub-communities of hilly- and paddy-converted farmlands compared to those in greenhouses (Tukey’s HSD test, *p* < 0.05, Figure 2f).

### 2.3. Environmental Adaptation of Abundant and Rare Sub-Communities

**Figure 3:**
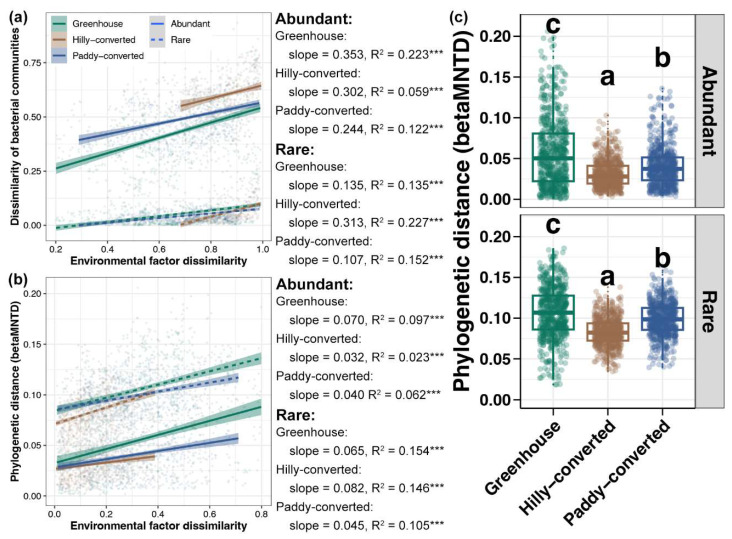
DDCS with environmental factors for the abundant and rare sub-communities among different cultivation modes based on the taxonomic (**a**) and phylogenetic (**b**) distances, respectively. *** represents the p-value of adonis test lower than 0.001. Lines in each sub-figure represents the fitted curve and gray shadow represents the 95% confidence interval. (**c**) Differences in the phylogenetic distance between abundant and rare sub-communities, respectively, between different soils from each cultivation mode. Different lowercase letters above each box in the same sub-figure represent significant differences among samples from different cultivation modes (Tukey’s HSD test, *p* < 0.05).

To explore the potential association between environmental conditions with the abundant and rare sub-communities in lemon farmlands, the DDCS was first performed. A significant DDCS with environmental factors was obtained for both abundant and rare sub-communities in all three cultivation modes (*p* < 0.05, Figure 3a). For abundant taxa, the strength of the DDCS was the strongest in greenhouses, followed by paddy-converted farmlands, and the weakest in hilly-converted farmlands, whereas this trend was just the reverse for rare sub-communities (Figure 3a). In addition to the DDCS based on taxonomic distance, the DDCS based on phylogenetic distance was further carried out. Similar to the results based on taxonomic distance, the DDCS with environmental factors based on phylogenetic distance was all significant for both abundant and rare sub-communities in all three cultivation modes (*p* < 0.05, Figure 3b). The trend of the DDCS strength among different cultivation modes was also consistent to the results of the DDCS based on taxonomic distance (Figure 3b). Moreover, variations in the phylogenetic distance of abundant and rare sub-communities between different soils from each cultivation mode were also compared. For both abundant and rare sub-communities, the phylogenetic distance between soils was the highest in greenhouse and the lowest in hilly-converted farmlands (Figure 3c), also consistent with the results based on taxonomic distance (Figure 2c).

**Figure 4:**
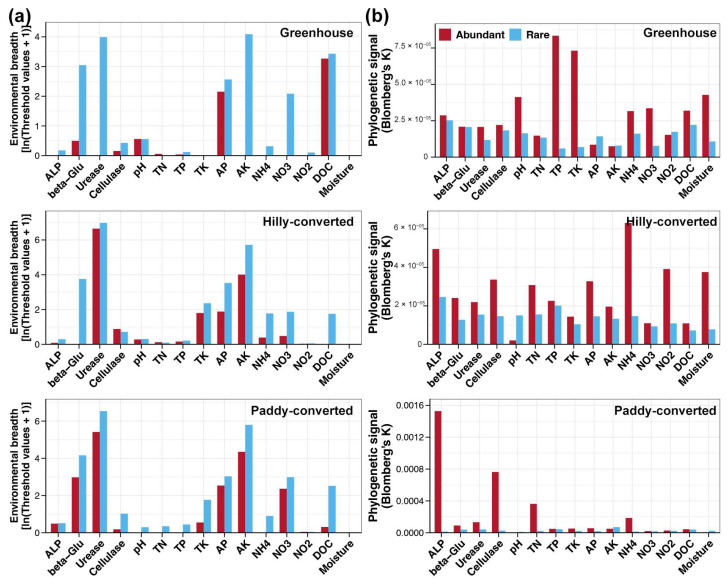
Environmental breadths (**a**) and phylogenetic signals (**b**) of abundant and rare taxa in response to environmental factors among different cultivation modes, respectively.

The environmental threshold analysis was further employed to compare the differences of abundant and rare sub-communities in response to each of the studied environmental factors. In all three studied cultivation modes, rare taxa exhibited a broader environmental breadth compared to abundant taxa for almost all measured environmental factors (Figure 4a). Moreover, relationships between the phylogeny of abundant or rare taxa with their environmental preferences were further measured. Stronger phylogenetic signals for most environmental factors were found in abundant taxa compared to those in rare taxa in all three cultivation modes (Figure 4b). This suggested that the narrow ecological preferences within the abundant sub-community could be due to their phylogenetic conservatism.

### 2.4. Community Assembly Mechanisms of Abundant and Rare Taxa

**Figure 5:**
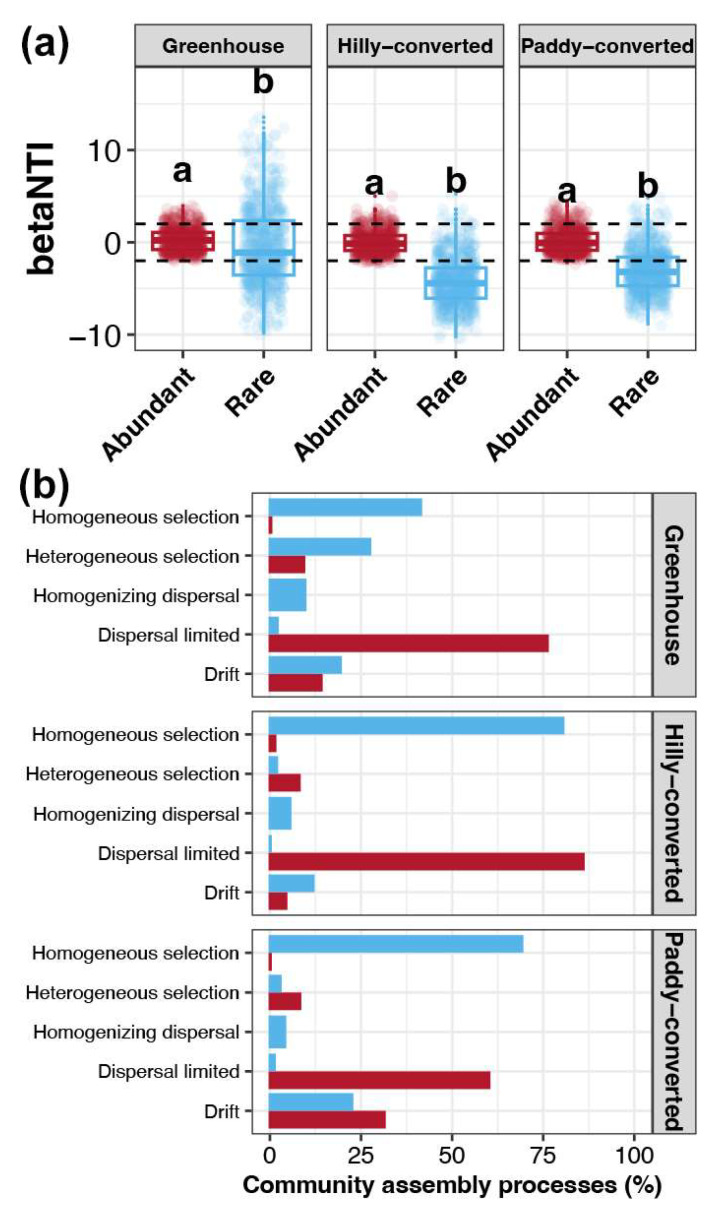
(**a**) Variations in betaNTI between the abundant and rare sub-communities in soils from each cultivation mode. Different lowercase letters above each box in the same sub-figure represent significant differences between abundant and rare sub-communities (Wilcox rank-sum test, *p* < 0.05). (**b**) Contributions of different ecological processes for assembly of abundant and rare sub-communities in soils from each cultivation mode. Red and blue colors represent the abundant and rare sub-communities, respectively.

Based on the results of the null model, the relative importance of stochastic and deterministic processes for the assembly of abundant and rare sub-communities among different cultivation modes was investigated. The median of betaNTI for abundant sub-communities in all three cultivation modes were between −2 to 2, indicating that stochastic processes governed their assembly (Figure 5a). In contrast, the betaNTI for rare sub-communities were significantly lower than that for abundant taxa in all three cultivation modes (Wilcox rank-sum test, *p* < 0.05), with the median lower than −2 in hilly- and paddy-converted farmlands (Figure 5a). These results suggested that deterministic processes contributed more to the assembly of rare sub-communities than those for abundant taxa. Dispersal limiting, a stochastic process, was the dominant ecological process for the assembly of abundant sub-communities in all three cultivation modes, while it was, instead of homogeneous selection, one of the deterministic processes in rare taxa (Figure 5b).

### 2.5. Associations of Environmental Conditions and Bacterial Community Assembly

**Figure 6:**
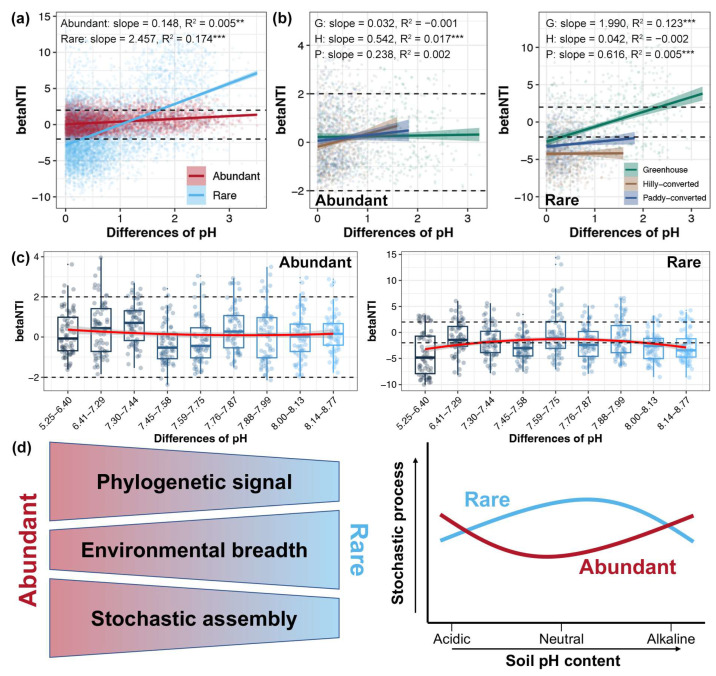
Relationships between betaNTI and differences in soil pH for abundant and rare sub-communities in all studied samples (**a**) and samples from different cultivation modes (**b**), respectively. ** and *** represent the *p*-value lower than 0.01 and 0.001, respectively. (**c**) Patterns of betaNTI across different categories in soil pH for the abundant and rare sub-communities. We ranked all 108 samples based on their pH from low to high; then, we sub-grouped them into 9 groups with 12 samples of each. Then, the pH intervals were determined according to the pH value belonging to each sub-group. (**d**) A conceptual paradigm showing environmental responses and stochastic processes in the assembly of abundant and rare bacterial sub-communities under the influence of soil pH in lemon farmlands.

Relationships between betaNTI and measured environmental factors were evaluated in order to explore the potential drivers for soil bacterial community assembly in lemon farmlands. Linear regression results showed that soil pH was the factor with the strongest relationships with assembly processes in both the abundant and rare sub-communities (Table 1). Although both of them were significant (linear regression, *p* < 0.05), the association between soil pH and betaNTI was stronger for rare sub-communities that that for abundant taxa (Figure 6a). These relationships were further explored in each cultivation mode, and significant associations were found in soil pH with abundant taxa in hilly-converted farmlands, while rare taxa were found in greenhouse and paddy-converted farmlands (Linear regression, *p* < 0.05, Figure 6b). Furthermore, the samples were separated into sub-groups based on the soil pH and correlated with betaNTI. With an increasing soil pH, the relative importance of stochastic assembly first decreased and then increased in the abundant sub-communities, while it first increased and then decreased in the rare-communities (Figure 6c).

## 3. Discussion

Comparing abundant and rare microbial taxa is essential for a deeper understanding of ecosystem complexity, and resilience, and the functional roles of microbes in environmental sustainability [38]. This comparison not only enhances our understanding of microbial ecology but also informs broader ecological and environmental management practices [39,40]. Based on the survey of soil bacteria in lemon farmlands with different cultivation modes, the present study provides evidence of the potential effects of the greenhouse on the soil bacteria community, indicating the lower richness but higher DDCS strength of abundant taxa. The results of this study also uncover disparate assembly processes controlling the abundant and rare sub-communities, which are mainly mediated by soil pH.

### 3.1. Effects of Cultivation Modes on Abundant and Rare Bacterial Sub-Communities

Soil bacterial communities play critical roles in agricultural productivity by decomposing organic compounds, recycling nutrients, and improving plant tolerance to stress [41]. Understanding the biogeography and distribution patterns of these soil bacterial communities can yield valuable insights underpinning sustainable agriculture. Moreover, the biogeographic patterns of different subdivisions of soil bacteria, rather than just at the whole community level, can provide a more nuanced view of microbial diversity and the response to environmental changes [42]. Previous studies have indicated that different agricultural land use practices (e.g., greenhouses, orchards, and paddy fields) drove distinct soil bacterial community structures and compositions [43]. The results of the present study also showed significant variations in the richness, proportion, and compositions of both abundant and rare bacterial sub-communities among soils of lemon farmlands with different cultivation modes (Figure 1 and Figure 2). The greenhouse was found to decrease the richness of soil bacterial communities compared to open-field cultivation due to the agricultural intensification and higher rates of nitrogen fertilization [44,45]. Our results further suggested that the decreased richness of soil bacterial communities in greenhouses was more obviously seen in abundant taxa (Figure 1b). In addition, a previous study indicated that the proportion of abundant taxa tended to increase with increasing environmental disturbance [29]. Generally, the disturbance from human activities is stronger in greenhouses compared to those open-field cultivation [46]. The highest total abundance of abundant bacterial taxa found in greenhouse soils in the present study (Figure 1c) consists of the previous discovery. Moreover, compared to rare taxa, abundant taxa generally have a lower beta-diversity—less variation in community composition compared to rare taxa [47]. A similar phenomenon was also observed in the present study with more close distribution patterns in abundant taxa compared to rare sub-communities (Figure 2a,b). These differences identified here could help monitor the impact of agricultural management on soil microbial communities.

Certain bacterial taxa are enriched in specific agricultural modes, which can serve as indicator species for monitoring soil health [48]. Preteobacteria, Chloroflexi, and Bacteroidota were found to be enriched in the abundant sub-communities of soils from lemon greenhouses compared to those open-field farmlands (Figure 2f). Proteobacteria have been associated with the production and absorption of greenhouse gases [49], making them an important component in understanding soil–climate interactions and the impacts of environmental change. Chloroflexi have been found to play important roles in the composting process and soil biota in sustainable agriculture [50]. Controlling the relative abundance of Chloroflexi has been shown to be closely related to reducing greenhouse gas emissions [51]. Bacteroidota are key degraders of complex organic matter in greenhouse soils, playing crucial roles in carbon cycling and nutrient turnover [52], and the application of biofertilizers can significantly decrease its relative abundance [53]. These enriched abundant bacterial phyla in soils of lemon greenhouses are valuable for developing biological monitoring tools for assessing the impacts of human activities.

Moreover, understanding the DDCS can help in predicting the distribution of microbes and assessing the impacts of human activities on microbial communities [54]. The overall similarity and rates of decay are primarily influenced by species abundances [55]. Abundant species are those that are common and widespread, while rare species are those that are infrequent and have limited distribution [56]. Thus, abundant species tend to show a more gradual DDCS due to their widespread distribution, while rare species may exhibit a steeper decay at short distances but have a weaker overall influence on the distance–decay relationship [57]. However, a steeper environmental-related DDCS was uncovered for abundant bacterial taxa in greenhouse soils compared to that for rare sub-communities in the present study (Figure 3a,b). The contradicting results between the previous and present studies could be due to the difference in geographic scales among the distinct investigations [58,59]. This study only focused on the lemon farmlands at a local scale and greenhouse buildings obviously limited the turnover of abundant soil bacteria due to their high abundance and wide distribution [60]. In contrast, the dispersal potential of rare taxa between different soil samples could be high based on their low frequency [61]. Understanding these patterns is crucial for biodiversity conservation, as it informs strategies for preserving species across different geographic scales.

### 3.2. Stronger Environmental Adaptations of Rare Bacterial Taxa

Microbial communities can exhibit resilience and resistance to disturbances, but the specific response depends on the nature and chronicity of the disturbance [62]. These responses can include shifts in community composition and function, which are critical for predicting how ecosystems adapt to long-term environmental changes [63]. Abundant and rare taxa have distinct ecological functions and environmental adaptations: comparing them can provide insights into the overall stability and functioning of microbial communities under environmental stresses or disturbances [64,65]. Generally, rare taxa are more sensitive to environmental changes and disturbances compared to abundant taxa, which have broader niches and can better adapt [66]. However, we observed a narrow environmental breadth of abundant bacteria taxa in soils of lemon farmlands compared to that of rare taxa (Figure 6d). The definition of abundant taxa having wider niches is gleaned more from previous studies on large geographic scales and spanning diverse ecosystems [67,68,69]. In this study, bacteria only need to have a high competitiveness in lemon farmlands to become abundant species, and a considerable portion of them do not have a broad environmental adaptability, so they will be classified as rare or intermediate taxa in larger scale studies. This inference is supported by the comparison of phylogenetic signals between abundant and rare sub-communities (Figure 6d). Lower phylogenetic signals indicated more closely related species in the abundant sub-communities, which generally exhibited more similar ecological preferences across environmental gradients [70,71]. Thus, the abundant taxa identified in this study were more specialized and sensitive to environmental changes. In contrast, rare taxa with lower phylogenetic signals are weaker in phylogenetic niche conservatism, which provide functional redundancy and contribute significantly to ecosystem multifunctionality [72,73]. This might explain the broader environmental breadths and distinct biogeographic patterns of rare taxa compared to abundant sub-communities among lemon farmlands with different cultivation modes.

### 3.3. Stochastic and Deterministic Processes Respectively Governing the Assembly of Abundant and Rare Bacterial Sub-Communities

Understanding the microbial assembly mechanisms of microbial communities is crucial for advancing our fundamental knowledge of microbial ecology [74]. Elucidating the relative importances of deterministic and stochastic processes in shaping microbial communities can guide strategies for managing and conserving microbial diversity in natural and engineered ecosystems [75]. Moreover, comparing the assembly processes between abundant and rare taxa provides insights into the distinct strategies they employ to adapt to environmental stresses [76]. Bacterial communities in greenhouses have been previously found to be more influenced by stochastic processes like dispersal limitation and less by deterministic processes like homogeneous selection, leading to a lower bacterial diversity and less closely associated taxa [77]. The findings of the present study further explored this perspective of stochastic processes contributing more to shaping the rare sub-communities in greenhouses (Figure 5a). In addition, similar to previous findings [10,30,31], the present study also revealed that abundant sub-communities are more affected by stochastic processes, including random events and dispersal limitations (Figure 6d). In contrast, rare sub-communities are generally more influenced by deterministic processes, which are driven by environmental selection pressures (Figure 6d). Dispersal involves the movement of microorganisms across different habitats, and selection is the process by which environmental pressures favor certain microbial traits [78]. This suggested that abundant taxa might be more restricted to turnover in soils of lemon farmlands and rare taxa are subjected to a greater convergence under environmental selection pressures.

### 3.4. Soil pH Mediating the Assembly Processes of Soil Bacterial Communities in Lemon Farmlands

On the basis of the above findings, determining the key factors that regulate the assembly processes of abundant and rare sub-communities in the soils of lemon farmlands is essential for stability and the functioning of soil ecosystems [79]. We determined that soil pH played a leading role in regulating soil bacterial community assembly with the stochastic ratio increased and decreased at the neutral pH for rare and abundant sub-communities, respectively (Figure 6d). Soil pH has also been recognized as the primary factor driving the distribution and function of microorganisms in farmland soils [57]. A study that collected arable soils with a broad pH range (4.26–8.43) revealed that soil pH was an important factor shaping the bacterial community composition, with a higher diversity in neutral samples and a lower one in acidic samples [53]. In addition, soil acidification, which was more pronounced in the greenhouse soils, was identified as a key driver mediating the changes in bacterial community assembly processes under agricultural intensification [77]. Moreover, bacterial growth is highly influenced by pH, with 50% of maximum growth at pH opt within an interval of ±1.7 pH units [80]. A soil pH closer to neutral might decrease the selective pressures and, consequently, reduce the effect of pH [81]. In this study, the decreased role of stochasticity in neutral environments may mean that adapted lineages of abundant taxa are reduced in neutral soils. The dominance of homogeneous selection in acid or alkaline soils suggests that rare taxa are more sensitive to an extreme pH; by contrast, the dominance of stochastic assembly in neutral soils implies the weaker niche-based exclusion and increased arrival of rare taxa lineages. Due to rare taxa being important contributors to microbial diversity [6], a changing soil pH might have a strong effect on microbial diversity.

Some agricultural management through targeted pH adjustment has been observed to optimize soil health and ecosystem functioning by leveraging pH–bacteria relationships. Liming acidic soils remains one of the most effective interventions for restoring bacterial equilibrium [82]. A longitudinal study demonstrated that lime applications increased soil microbial diversity and enhanced some bacteria to accelerate nutrient cycling [83]. In addition, field trials in China’s North China Plain revealed that maintaining a soil pH between 6.2–6.8 optimized the bacterial richness, enabling functional redundancy during environmental perturbations [84]. Moreover, alleviating soil acidification suppressed bacterial wilt disease by inhibiting *Ralstonia solanacearum* by recruiting potentially beneficial rhizobacteria [85]. However, it should be noted that the nonlinear response of bacterial diversity to pH necessitates region-specific management [86]. By embracing pH as an ecological lever rather than a mere chemical metric, farmers could unlock the full potential of soil as a living, dynamic resource.

## 4. Materials and Methods

### 4.1. Sample Collection

Soil samples were collected from farmlands with more than five years of lemon cultivation at the Chongqing Tongnan Seedling Breeding Center located in the Guopo Village, Baizi Town, Chongqing, China. One-hundred and eight soil samples were collected from nine farmlands with twelve samples in each. A multi-point sampling method was used to collect soils with surface bulk soils at three random points within a 20 cm radius around a lemon trunk, and the collected soils were mixed into one sample. Each soil sample was divided into two parts: one was for the measurement of the soil properties, and another part was treated with liquid nitrogen and then cryopreserved at −80 °C for sequencing of soil bacterial communities. Among the nine farmlands, three of them were transformed from hilly areas (Hilly-converted), three of them were transformed from paddy fields (Paddy-converted), and the last three are orchards with greenhouse (Greenhouse). This sampling strategy allowed us to explore whether agro-ecosystems with different backgrounds exhibited similar trends.

### 4.2. Measurements of Environmental Factors

Edaphic variables, including moisture, pH, ammonium-nitrogen, nitrite-nitrogen, nitrate-nitrogen, total nitrogen (TN), available phosphorus (AP), total phosphorus (TP), available potassium (AK), total potassium (TK), and dissolved organic carbon (DOC), were measured using standard analytical methods [87,88]. Meanwhile, the soil enzyme activities, including urease, cellulase, alkaline phosphatase (ALP), and beta-glucosidase (beta-Glu), were determined using respective ELISA kits (Shanghai Jiwei Biological Technology Co., Ltd., Shanghai, China) using an RT-6100 spectrophotometer (Rayto Life and Analytical Sciences Co., Ltd., Shenzhen, China).

### 4.3. High-Throughput Sequencing and Data Processing

After DNA was extracted by the FastDNA^®^ SPIN kit for soil (MP Biomedicals, Santa Ana, CA, USA), bacterial community was determined using high-throughput sequencing of the V3–V4 region of 16S rRNA gene with the primer pair 341F (GCCTCCCTCGCGCCATCAGCAGTAGACGT) and 806R (GCCTTGCCAGCCCGCTCAG) [89] on a NovaSeq 6000 platform (Illumina Inc., San Diego, CA, USA). A negative control was used throughout the whole-sequencing process and no obvious microbial contamination was found. Based on the unique barcode at the end of reverse primer for each gene, sequenced reads were assigned to the corresponding samples. Then, sequences were filtered for quality (reads with average Phred scores > 20, no ambiguous bases or mismatches in the primers, homopolymer runs > 8, and sequence lengths > 250 bp), paired-end reads were assembled, and chimera was removed using the QIIME2 (Quantitative Insights Into Microbial Ecology 2) program [90]. The remained reads were clustered into amplicon sequence variants (ASVs) based on the DADA2 algorithm [91] and the representative sequences were classified within the SILVA database (release 138) [92]. By a randomly selected subset of 24,168 sequences from each sample to standardize the sequencing effort across samples, the ASV abundance table was obtained. ASVs with average relative abundance above 0.1% and below 0.01% across all samples were recognized as “abundant” and “rare” taxa [67].

### 4.4. Statistics Analysis

All statistics analyses were performed in R v4.4.2 and the results were visualized by the “ggplot2” package (v3.5.1). Richness (Chao1 index) of abundant or rare sub-communities in each sample and the Bray–Curtis distance of them between different samples were calculated by the “vegan” package (v2.6-10) [93]. Tukey’s HSD test (“multcomp” package) was used to assess differences in the richness, total abundance, and Bray–Curtis distance of abundant or rare sub-communities, respectively, among farmlands with different cultivation modes [94]. Principal coordinates analysis (PCoA) and adonis test based on the Bray–Curtis distance of abundant or rare sub-communities were also achieved, respectively, by the “vegan” package. Differences in the relative abundances of dominant bacterial phyla between the abundant and rare sub-communities and variations of them in abundant or rare sub-communities among different cultivation modes were further compared by the Tukey’s HSD test.

Beta mean nearest taxon distance (betaMNTD) metric of abundant and rare sub-communities was, respectively, calculated by the “picante” package (v1.8.2) [95], and their differences in samples among different cultivation modes were evaluated by Tukey’s HSD test. Euclidean distance of measured environmental factors between each of two samples were calculated (“vegan” package) and the distance–decay community similarity (DDCS) was assessed as the slope of an ordinary least-squares regression between it with the Bray–Curtis distance or betaMNTD of abundant or rare sub-communities. In addition, the environmental breadth of abundant or rare sub-communities in response to each of the environmental factors was estimated by the threshold indicator taxa analysis (“TITAN2” package) (v2.4.3) [96]. Then, the phylogenetic signals of abundant and rare sub-communities to measured environmental factors were appraised by the Blomberg’s K statistic (“picante” package) (v1.8.2) [95].

A null model method reported by Stegen et al. [97] was used to investigate the assembly processes of abundant and rare sub-communities. Based on the value of beta nearest taxon index (betaNTI) and Bray–Curtis-based Raup–Crick metric obtained from the null model, the relative importance of ecological processes for community assembly were estimated [98]. For betaNTI, values < − 2 indicate homogeneous selection, whereas values > 2 indicate heterogeneous selection. The Raup–Crick metric was used to partition the remaining parts with |betaNTI| ≤ 2. Here, Raup–Crick < − 0.95 represents homogenous dispersal, Raup–Crick > 0.95 represents dispersal limitation, and |Raup–Crick| ≤ 0.95 represents ecological drift. Then, the major factors that influenced the assembly processes of abundant and rare sub-communities were assessed using the linear regression comparing betaNTI values with the Euclidean distance matrices of each of the environmental factors.

## 5. Conclusions

Based on the investigation of soil bacterial communities in diverse lemon farmlands, we observed the distinct diversity, composition, and assembly of abundant and rare sub-communities. Meanwhile, significant effects of cultivation modes, especially for greenhouses, on both abundant and rare bacterial sub-communities were observed. A lower richness, higher beta diversity distance, and stronger DDCS of abundant sub-communities were found in greenhouses compared to other open-field farmlands. Moreover, we proposed a conceptual paradigm describing the assembly of abundant and rare bacterial sub-communities influenced by environmental factors in lemon farmlands (Figure 6d). The abundant sub-communities were more phylogenetically closely clustered but less adaptable to environmental changes than the rare taxa, which were more governed by stochastic assembly. Soil pH regulated the assembly of both abundant and rare sub-communities. A neutral pH led to an increase in stochastic assembly in the rare sub-communities, while it led to a decrease in abundant taxa. These results are beneficial to the understanding of the maintenance of microbial diversity and their related ecosystem functions in lemon farmlands, and should be considered for the development of strategies for ecosystem management.

## Figures and Tables

**Table 1:** Linear regression between differences in environmental factors with betaNTI of abundant or rare sub-communities.

Environmental Factors	Abundant Taxa			Rare Taxa		
	Slope	*R* ^2^	*p*-Value	Slope	*R* ^2^	*p*-Value
ALP	0.1603	0.0060	0.0005	0.5656	0.0088	2.64 × 10^−5^
beta-Glu	0.0028	0.0125	6.28 × 10^−7^	0.0008	−0.0004	0.6260
Urease	5.72 × 10^−6^	−0.0005	0.9250	9.87 × 10^−5^	−0.0004	0.5809
Cellulase	0.0966	0.0080	5.97 × 10^−5^	0.0272	−0.0005	0.7014
pH	0.1484	0.0050	0.0012	2.4570	0.1745	6.72 × 10^−81^
TN	0.0770	−0.0003	0.5100	2.0898	0.0190	1.03 × 10^−9^
TP	−0.1068	0.0007	0.1348	−0.6283	0.0042	0.0028
TK	0.0015	−0.0005	0.8607	−0.0173	−0.0003	0.4852
AP	−0.0009	6.94 × 10^−5^	0.2877	0.0055	0.0022	0.0241
AK	−0.0006	0.0060	0.0004	−0.0008	0.0008	0.1168
NH4	0.0465	0.0038	0.0043	0.2238	0.0110	2.80 × 10^−6^
NO3	0.0037	0.0006	0.1378	−0.0393	0.0145	8.98 × 10^−8^
NO2	0.9222	0.0009	0.0960	18.1326	0.0650	1.19 × 10^−29^
DOC	0.0038	0.0006	0.1463	0.0472	0.0195	6.64 × 10^−10^
Moisture	3.0480	0.0024	7.38 × 10^−7^	32.0664	0.1645	5.54 × 10^−76^

## Data Availability

All raw sequences of sediment bacterial communities studied in this study have been submitted to the NCBI Sequence Read Archive (SRA) database under the BioProject number PRJNA1100232.
